# Hypofractionated Radiotherapy for Anaplastic Thyroid Cancer: Systematic Review and Pooled Analysis

**DOI:** 10.3390/cancers12092506

**Published:** 2020-09-03

**Authors:** Dmytro Oliinyk, Teresa Augustin, Viktoria Florentine Koehler, Josefine Rauch, Claus Belka, Christine Spitzweg, Lukas Käsmann

**Affiliations:** 1 Department of Radiotherapy and Radiation Oncology, University Hospital, LMU Munich, 81377 Munich, Germany; Dmytro.Oliinyk@med.uni-muenchen.de (D.O.); Teresa.Augustin@med.uni-muenchen.de (T.A.); Josefine.Rauch@med.uni-muenchen.de (J.R.); Claus.Belka@med.uni-muenchen.de (C.B.); 2 Department of Internal Medicine IV, University Hospital, LMU Munich, 81377 Munich, Germany; viktoria.koehler@med.uni-muenchen.de (V.F.K.); Christine.Spitzweg@med.uni-muenchen.de (C.S.); 3 German Cancer Consortium (DKTK), Partner Site Munich, 80336 Munich, Germany

**Keywords:** ATC, anaplastic thyroid cancer, hypofractionated, irradiation, survival

## Abstract

**Simple Summary:**

Anaplastic thyroid carcinoma is an aggressive cancer subtype with a dismal prognosis. Multimodal treatment approaches consisting of surgical resection, radiation therapy (RT) and chemotherapy have resulted in longer overall survival and promising outcomes. Hypofractionated RT is an alternative to conventional RT regimens. In this study, we aim to evaluate the outcome of hypofractionated regimens, perform a systematic review concerning hypofractionated RT and pooled analysis of this treatment modality. Hypofractionated RT appears to be non-inferior compared to conventional RT concerning OS after propensity score matching. In addition, radiation dose escalation correlated with a longer OS. In conclusion, hypofractionated RT is effective with manageable toxicity and could be an integral part in multimodal treatment.

**Abstract:**

Anaplastic thyroid carcinoma (ATC) is associated with a poor prognosis due to aggressive tumor growth and high treatment resistance. Hypofractionated treatment concepts may be more effective and less time consuming compared to normofractionated radiotherapy (RT). In this retrospective study, we aim to evaluate the outcome of hypofractionated regimens and perform a systematic review concerning hypofractionated RT and pooled analysis of this treatment modality. A systematic review using the MEDLINE/Pubmed and Cochrane databases was performed. Data from all eligible studies were extracted, and a pooled analysis of literature and our cohort (*n* = 60) was carried out to examine patient characteristics, toxicity, and outcomes of patients with ATC. As a result, median overall survival (OS) of the single center cohort was four (range 1–12) months. Survival rates at one, three, and six months were 82%, 55%, and 36%, respectively. In univariate analyses, multimodal treatment (*p* = 0.006) and gender (*p* = 0.04) were correlated with an improved OS. Six studies with a total number of 152 patients undergoing hypofractionated RT treatment were analyzed. The pooled analysis included four patient cohorts with 60 patients and showed median OS of 5.3 (range: 1–24) months. Multimodal treatment (*p* < 0.001) and a cumulative radiation dose ≥50 Gy in equivalent dose in 2 Gy fractions (EQD2) (*p* = 0.014) correlated with an improved OS. On multivariate analysis, multimodal treatment (*p* = 0.003, hazard ratio (HR): 0.636, 95% confidence interval (CI): 0.469–0.861) was an independent predictor for longer OS. After propensity score matching (PSM), hypofractionated RT appears to be non-inferior compared to normofractionated RT concerning OS. In conclusion, hypofractionated RT is effective with manageable toxicity. A dose escalation with ≥50 Gy (EQD2) correlated with a longer OS. Hypofractionated RT could be an integral part in multimodal treatment with a promising outcome.

## 1. Introduction

Anaplastic thyroid carcinoma (ATC) remains one of the rarest and most aggressive neoplasms of the thyroid gland, enumerating a relatively stable incidence of approximately 3.4% in Europe [1]. ATC confers a dismal prognosis due to rapid progression with a median overall survival (OS) of 3–6 months [2]. Current treatment modalities incorporate multimodality approaches including surgery, radiotherapy (RT), and chemotherapy, as well as novel systemic treatment approaches with increasing research on targeted therapies including druggable BRAF V600E or RAS mutations, RET, ALK or NTRK fusions, and PD-L1 overexpression [3,4]. Depending on resectability and stage of disease, surgery with adjuvant chemoradiotherapy or definitive RT with concurrent chemotherapy (ChT) (usually with doxorubicin or platinum-based agent) can be considered standard of care [5,6]. Quality of life (QoL) and locoregional control represent primary treatment goals and need to be taken into account for decision making. Patients’ overall prognosis should be considered when tailoring the treatment regimen. With the aim of a personalized treatment approach, patients with a limited prognosis should preferably receive a short palliative regimen consuming as little of the patients’ remaining lifespan as possible.

To date, several established fractionation regimens can be administered in patients with ATC. Conventional irradiation once daily with 2 Gy per fraction up to 70 Gy of total dose was used for ATC treatment as an established standard option according to National Comprehensive Cancer Network (NCCN) Clinical Practice Guidelines in Oncology [5] and American Thyroid Association Guidelines [7]. Historically, altered fractionation techniques, e.g., hyperfractionated RT, have been introduced but failed to achieve less toxicity or improved outcome [6,8,9,10]. Delivery of higher radiation doses per fraction over a shorter period of time in form of hypofractionated RT could theoretically have advantages in terms of quality of life (QoL) and achieving local control (LC). In the preclinical study of Oweida et al. [11], hypofractionated RT demonstrated enhanced local tumor control compared to normofractionated RT in a mouse model. In addition, several clinical studies found promising results concerning hypofractionated RT in the treatment of ATC [12,13,14]. The aim of the present study is to evaluate the outcome and toxicity of hypofractionated regimens in the treatment of ATC at our tertiary care center and to perform a systematic review of literature with a pooled data analysis.

## 2. Results

### 2.1. Single Center Evaluation

#### 2.1.1. Treatment

A total of 17 ATC patients treated with hypofractionated RT at out center were identified. We excluded all patients treated in palliative intention and with a cumulative radiation dose ≤30 Gy. The remaining patients (*n* = 11) were included in the analysis. Total thyroidectomy was performed in three patients (27%), respectively, before irradiation. ChT was administered in six patients (55%), four patients (67% of ChT group) received ChT (carboplatin AUC 2 with Paclitaxel 50mg/m^2^ or doxorubicin (10 mg/m^2^) weekly) in combination with irradiation, while two patients (33% of ChT group) received ChT in a neoadjuvant concept with doxorubicin or carboplatin/paclitaxel before irradiation. Irradiation was administered using three-dimensional conformal RT (3D-CRT) technique in eight patents (73%), and three patients (27%) were treated using intensity modulated radiation therapy (IMRT). All patients were treated with single dose of 2.50 Gy (18%) or 3.00 Gy (82%). The cumulative radiation dose was calculated in equivalent dose in 2 Gy fractions (EQD2). The median EQD2 of our cohort was 49 (range 32–55) Gy (Table 1).

#### 2.1.2. Outcome

**Figure 1:**
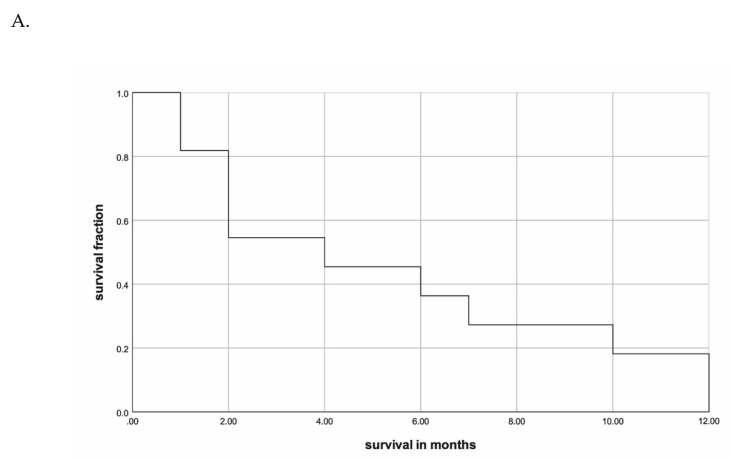

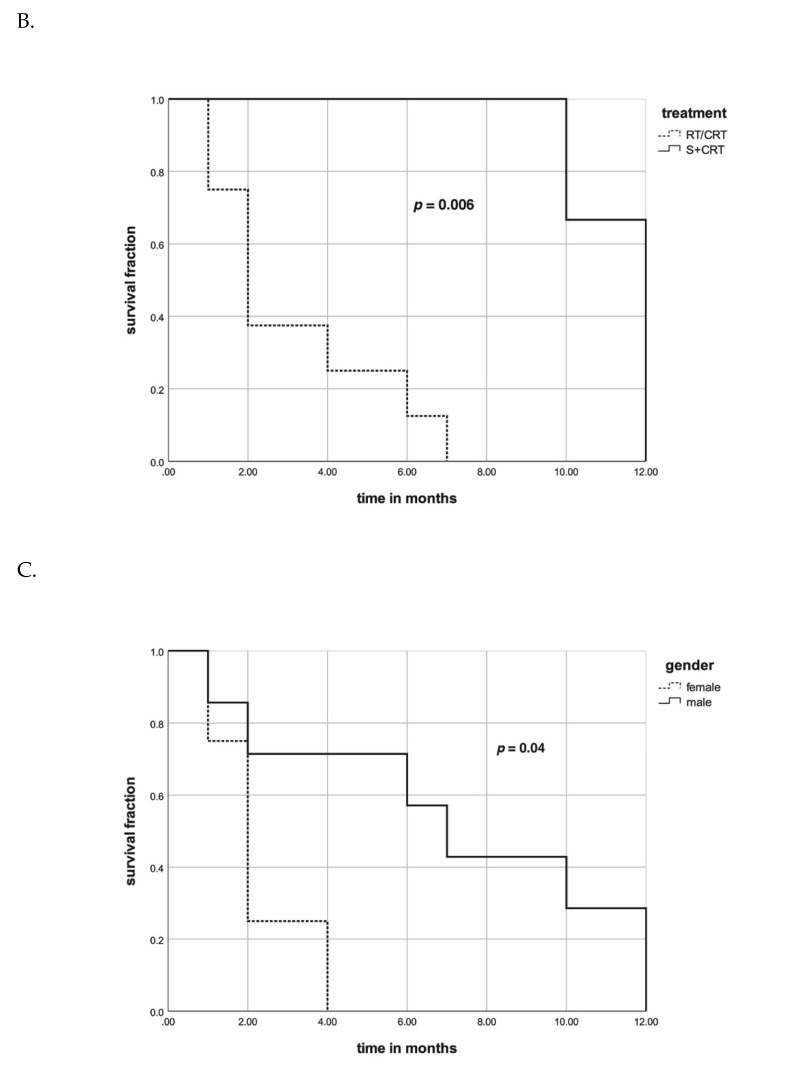
(**A**) Kaplan–Meier curve concerning overall survival of the single center cohort. (**B**) Kaplan–Meier curves concerning treatment mode for overall survival in the single center cohort. The *p*-value was calculated with the log-rank test. (**C**) Kaplan–Meier curves concerning gender for overall survival in the single center cohort. The *p*-value was calculated with the log-rank test.

Median OS of the single center cohort was 4 (range 1–12, 95% confidence interval (CI): 0.763–7.237) months. Survival at one, three, and six months was 82%, 55%, and 36%, respectively (Table 2, Figure 1A). No local progression was observed during RT or within follow up. In univariate analyses, multimodal treatment (*p* = 0.006) and gender (*p* = 0.04) correlated with an improved OS (Table 2, Figure 1B,C), respectively. On multivariate analysis for OS no factor achieved significance. Age (*p* = 0.106), Eastern Cooperative Oncology Group (ECOG) performance status (*p* = 0.326), and RT technique (*p* = 0.701) were not associated with OS. 

#### 2.1.3. Treatment-Related Toxicity

Adverse events were evaluated according to the Common Terminology Criteria for Adverse Events (CTCAE) Version 4. The most frequent side effects of local radiation were dysphagia, dysphonia, dermatitis, and mucositis. Grade 3 acute toxicities of dysphagia, dysphonia, dermatitis, and mucositis were observed in 18%, 18%, 9%, and 9% of patients, respectively. Therapy-related toxicity grade 4/5 was not observed.

### 2.2. Systematic Review

**Figure 2:**
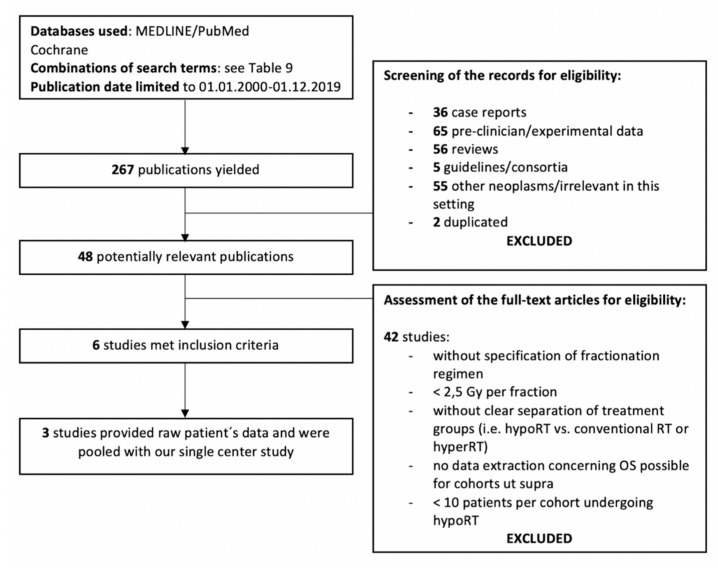
A PRISMA (Preferred Reporting Items for Systematic Reviews and Meta-Analyses) flowchart for systematic review of literature with results summary.

In total, 267 studies were yielded by an initial literature search (MEDLINE/PubMed). Evaluation of the Cochrane database did not provide any eligible data. In total, 261 publications were manually excluded after abstract and full-text screening. Fifty-six of the excluded papers were reviews and hence inspected for relevant citations. All of the cited studies on hypofractionated RT were excluded due to the publication dates not meeting inclusion criteria. After abstract screening, 219 studies were excluded for reasons shown in Figure 2. A total of 48 publications was selected for full-text analysis. Six publications met inclusion criteria and were included in the systematic review (Figure 2; [12,13,14,15,16,17]). Included publications involved patient cohorts with heterogeneous stage distribution ranging from 26 to 62 patients [12,13,14,15,16,17]. Hypofractionated RT was administered to a total of 152 patients with at least 43% of all patients diagnosed in UICC stage IVC. Characteristics of patients, treatment modalities, symptoms, outcome, and toxicities that were reported in the included studies are shown in Table 3, Table 4, Table 5 and Table 6. 

Four studies [14,15,16,17] reported patient symptoms at initial diagnosis, but only Wang et al. [16] specified the symptoms of the cohort administered hypofractionated RT. Apart from the most common symptom of neck mass (73–88%), several impairments have been reported, including dysphagia (17–54%), dysphonia (31–50%), dyspnea (20–33%), and other [12,16]. Surgery and ChT was reported in 22–82% and 14–88% of hypofractionated RT treated patients, respectively. The median prescribed total dose was ≤54 Gy with the median dose per fraction ranging from 3 Gy to 5 Gy. Median OS was 3–9.3 months. Two authors reported a relatively long median OS of 9.3 and 6 months, respectively [12,14]. Survival rates at three, six, and 12 months were reported or calculated according to data of four authors [12,13,16,17]. Remarkably, a patient cohort of Stavas et al. [12] stands out with a survival rate at 12 months of 41.2%. Local recurrence rate ranged from 18% up to 29%. 

### 2.3. Pooled Data Evaluation

Individual patients’ data of three cohorts [12,13,17] met our database assessment protocol and were, therefore, pooled with our single center cohort (*n* = 71) for further evaluation. After exclusion of palliative radiation with a cumulative radiation dose <30 Gy (EQD2), pooled analysis included a total of 60 patients treated with hypofractionated RT. Median age was 73 (range 49–92) years, 42% showed ECOG ≥2, and 50% of patients presented with distant metastases at initial diagnosis. Furthermore, 60% in the pooled cohort received an EQD2 dose of hypofractionated RT ≥50 Gy. Single dose ranged from 2.50 Gy to 5.00Gy in the pooled patient cohort. Concurrent ChT was administered in 62% of patients and 42% underwent either total or partial thyroidectomy. 

**Figure 3:**
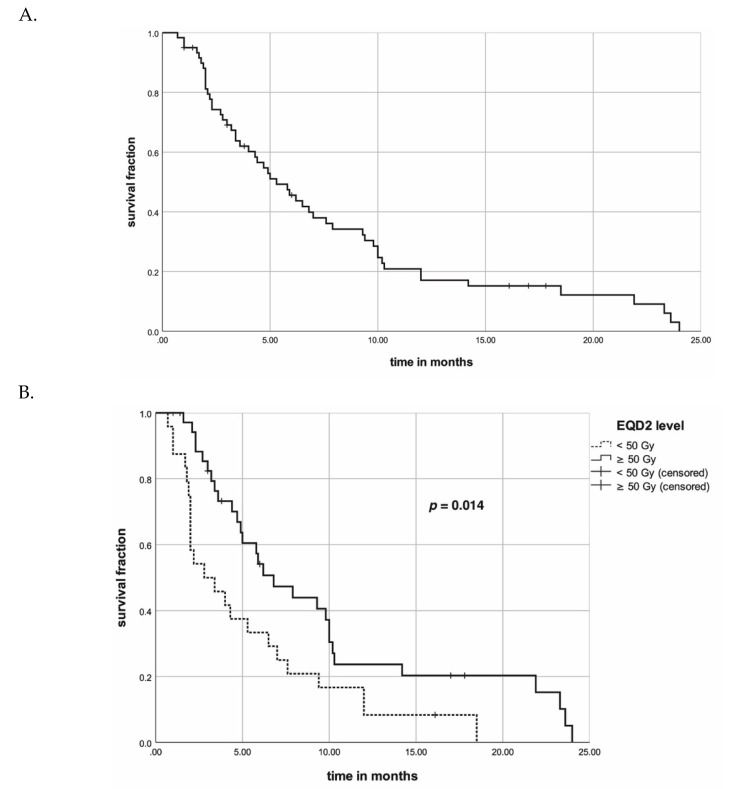

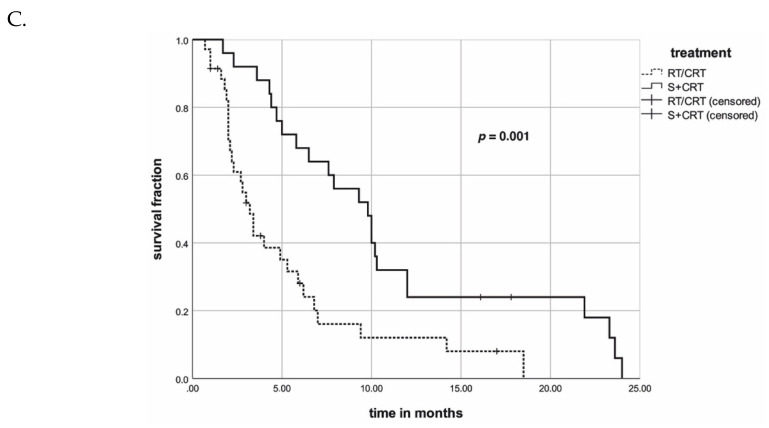
(**A**) Kaplan–Meier curve for overall survival of pooled patient cohort. (**B**) Kaplan–Meier curves concerning EQD2 level for overall survival of pooled patient cohort. The *p*-value was calculated with the log-rank test. (**C**) Kaplan–Meier curves concerning multimodal treatment for overall survival of pooled patient cohort. The *p*-value was calculated with the log-rank test.

Median OS of the pooled patient cohort was 5.3 (range: 1–24, 95% CI: 3472–7128) months. Survival at three, six, and 12 months were 69%, 46%, and 17%, respectively (Figure 3A). 

In univariate analysis, EQD2 dose in exceed of 50 Gy (*p* = 0.014) and administration of multimodal treatment (surgery and chemoradiotherapy (S + CRT), *p* < 0.001) correlated with an improved OS (Table 7, Figure 3B,C), respectively. A trend for improved survival was found in younger age (<73 age, *p* = 0.068) and a single dose level of 2.5–3.5 Gy (*p* = 0.077). On multivariate analysis, multimodal treatment (*p* = 0.003, hazard ratio [HR]: 0.636, 95% confidence interval [CI]: 0.469–0.861) were significantly associated with an improved OS, whereas a higher EQD2 >50 Gy (*p* = 0.065) did not achieve significance on multivariate analysis.

### 2.4. Propensity Score Matching (PSM)

Individual patients´ data of three cohorts [12,13,17] met our database assessment protocol, and our single center cohort were included in the propensity score matching (PSM) analysis. Normofractionated RT was defined as a single dose of less than 2.5 Gy and hypofractionated RT with ≥2.50 Gy. Patients receiving palliative radiation with ≤30 Gy were excluded from evaluation. Patients treated with normofractionated RT were matched in a 1:2 ratio to patients treated hypofractionated RT. To each patient treated with normofractionated RT, two corresponding patients with exactly the same ECOG PS and gender were matched. PSM also considered age and treatment mode. Eighteen normofractionated patients were matched to 36 hypofractionated patients (Table 8). In the normofractioanted subgroup, 83% of all patients were treated with a single dose of 2 Gy and the median cumulative radiation was 60 Gy (range: 44–71, EQD2). In the hypofractionated subgroup, median cumulative radiation dose was 55 Gy (range: 33–65, EQD2). 

**Figure 4:**
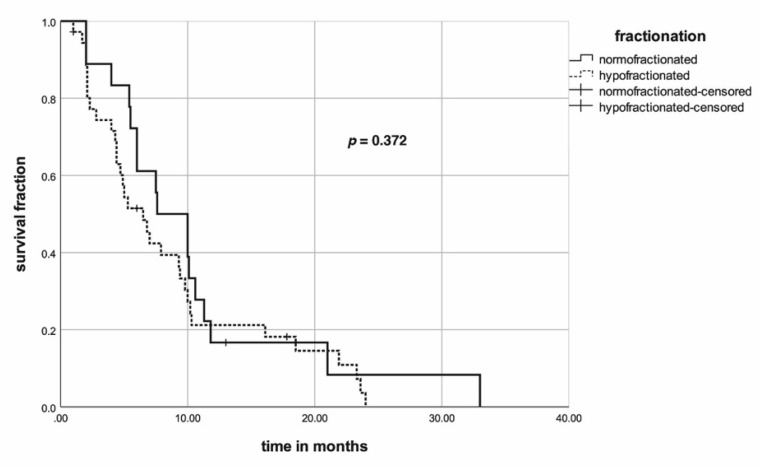
Kaplan–Meier curves concerning fractionation regimen for overall survival of PSM cohort. The *p*-value was calculated with the log-rank test.

Median OS of the entire PSM cohort was seven months (range: 1–33) with six, 12, and 24 months survival rates of 55%, 20%, and 3%. Median OS of the normofractionated RT subgroup was eight months (range: 1–33) with six, 12, and 24 months survival rates of 61%, 17%, and 8%. Median OS of the hypofractionated RT subgroup was seven months (range: 1–24) with six, 12, and 24 months survival rates of 52%, 21%, and 0%. Fractionation regimen achieved no significance (*p* = 0.372) in univariate analysis for OS (Figure 4).

## 3. Discussion

We report on the utilization of hypofractionated RT in a pooled patient cohort of 71 patients with ATC. To our knowledge, this is one of the largest studies reported to date, evaluating hypofractionated RT that was defined as a single dose per fraction ≥2.5 Gy [12,13,17]. 

The outcomes concerning OS and treatment-related toxicity reported in our pooled analysis are consistent with previous reports, with the majority of ATC patients presenting with symptomatic or metastatic disease. Improved OS in our cohort was observed in patients receiving multimodal treatment (*p* = 0.006) and male patients (*p* = 0.04). Administering ChT concurrent to hypofractionated RT showed a survival benefit of more than 30% at 12 months but was not an independent predictor (*p* = 0.327). The results of the pooled data analysis suggest that a total dose of EQD2 ≥50 Gy (*p* = 0.014) and multimodal treatment (*p* < 0.001) correlate with longer survival and, hence, are crucial for favorable OS. 

When applied in ATC, conventional RT has been shown to provide symptom palliation with similar outcomes compared to conventional RT regarding local control [6,12,13,17]. In this context, Oweida et al. [11] investigated radiosensitivity toward hypofractionated RT of human ATC cell lines in an orthotopic mouse model [11,18] following the in vitro characterization of the levels of radiosensitivity based on genetic profiling of the ATC cell lines. The definition of hypofractionated RT at ≥2.5 Gy per fraction is aligned with our treatment protocol. A 51.8-fold decrease in local tumor growth (*p* = 0.0097) assessed by average photon radiance (*p* = 0.0094) in vivo at day 36 was reported in that study compared to the control, whereas conventional RT showed a 6.7-fold decrease (*p* = 0.0057), respectively. In addition, hypofractionated RT treated mice had significantly longer OS than conventionally irradiated mice (HR = 6.049, 95% CI 1.863–28.05, *p* < 0.001) and a decreased rate of pulmonary metastases (*p* < 0.001), resulting in a strong preclinical rationale for the utilization of hypofractionated RT concepts.

To date, however, the use of hypofractionated RT in the treatment for ATC remains highly controversial. It is still mostly administered in palliative setting with a common cumulative dose ≤30 Gy [6]. Despite extensive research, studies comparing differently fractionated RT regimens for ATC are not available. For this purpose, we have investigated hypofractionation as an integral part of ATC treatment. Due to its rapid progression and the early onset of metastatic disease, management of ATC patients requires a multimodal approach including surgical resection of the primary tumor followed by chemoradiation [5,6,7]. 

The most recent study on ATC of Fan et al. [19] provided a comprehensive retrospective analysis of different outcomes in 104 ATC patients treated in a multimodal approach, which was administered to a total of 51% of patients and had a significant association (*p* = 0.017) with a decreased risk of local disease progression, but no association with OS was found. Multimodal treatment approaches such as surgery followed by concurrent chemoradiotherapy have been shown to be significantly relevant for the beneficial OS as an independent predictor by Glaser et al. [20]. Similar findings were reported by several authors [12,20,21,22,23] and may be attributable to lower recurrence rate [24] and a decrease in local complication rate caused by impending of trachea or damage to esophagus and carotid artery [23,25]. Our single center data as well as our pooled analysis supports the multimodal treatment approach (*p* = 0.006, *p* < 0.001).

However, normofractionated RT remains the standard care in these studies, and data evaluating other fractionation regimens such as hypofractionation are limited [20,21,22,23,26]. Conversely, studies gathered by systematic review investigated the integration of hypofractionation into the treatment. Nachalon et al. [14] investigated hypofractionated RT in 23 patients with ATC (surgical resection performed in 22%) and reported ChT to have a significant effect on survival (*p* = 0.01; administered to 48% of all patients). Stavas et al. [12] applied hypofractionated RT to 17 ATC patients in combination with surgery (82%) and ChT (88%-paclitaxel with or without carboplatin). Notably, Stavas et al. [12] and Nachalon et al. [14] do also stand out with their reported survival rates of 9.3 (range: 4.6–14) and 6 (range 2.1–9.8) months, respectively. These OS-rates are comparable to what has been reported for the entire cohort by e.g., Fan et al. [19] (seven months: 95%; CI: 4.5–9.5 months), where hyperfractionation and conventional fractionation regimens were used instead. Therefore, integration of hypofractionated RT into multimodal treatment could be considered for patients with ATC.

Apart from multimodal approaches, one of the crucial findings concerning radiotherapy for ATC was a significant dose-response relation [20,26,27]. Wendler et al. [26] showed the dose-response relation in a multi-center study for a cohort consisting of 100 patients with a total EBRT > 40 Gy (HR  =  0.34, 95% CI 0.15–0.76, *p* = 0.008).

Large-scale analyses of patients from National Cancer Data Base by Glaser et al. [20] or Pezzi et al. [27] showed improved OS to be associated with high-dose RT in exceed of 59.3 Gy (HR = 0.67, *p* < 0.005) and 59 Gy (*p* = 0.008), respectively. These results are comparable to our findings in both, single center and pooled data evaluation with an EQD2 > 50 Gy being associated with longer OS. Importantly, data of Nachalon et al. [14] on the beneficial outcome of ATC patients treated with hypofractionated RT in curative intention (*p* < 0.001) possibly implies comparable dose-response relation given different irradiation dosages of the gross tumors (70 Gy vs. 50–63 Gy vs. <30 Gy). Nevertheless, decisions for specific treatments were made based on individual characteristics of patients, including performance status, disease progression and resectability. Compared to our data, Takahashi et al. [13] similarly reported a total dose of >50 Gy (*p* = 0.049) to correlate with longer OS in the univariate analysis. In order to compare normofractionated to hypofractionated RT, we performed a PSM analysis based on the same database assessment protocol with a 1:2 matching. After exclusion of palliative treatment and adjustment for performance status and gender, hypofractionated RT appears to be non-inferior to normofractionated RT concerning OS. Although, ATC is thought to be relatively radioresistant, treatment response could be achieved with sufficient cumulative radiation doses using hypofractioned regimes.

Development of distant metastases is a common part of disease progression in patients with ATC and can thus be a limiting factor for therapy related decisions. It is considered a significant risk factor for the survival [28]. Correspondingly, Stavas et al. [12] demonstrated a difference in the median OS for those patients with and without distant metastases (6.4 months vs. 14.2 months), respectively. This is also comparable with the results on metastatic status that were found in the studies mentioned previously [20,21,26,27]. In contrast, however, Wang et al. [16] and our study found no impact of TNM stage on progression-free survival or OS.

In addition, Quality of life (QoL) remains one the most important therapy goals in ATC and sufficient palliation of symptoms impacts OS and PFS. Indeed, Sugitani et al. [28] evaluated 677 patients with ATC from 38 different institutions and identified presence of acute local symptoms, such as severe dysphonia, dysphagia, dyspnea, and progressive tumor growth <1 month (*p* = 0.0014), as significant risk for shorter OS in both univariate and multivariate analysis. Correspondingly, hypofractionation was reported to achieve local control in 71% of patients in an Australian study of So et al. [17]. It was administered to a cohort of 14 patients, who had a distant metastatic disease in 50%. In total, several studies [12,13,16,17] gathered by systematic review showed that hypofractionation can sufficiently provide an acceptable local control rate of 71–82% at the time of the last follow-up or death. This is comparable to the study mentioned previously [19] and not inferior to the results of single or combined modality treatment of Veness et al. [29]. In our study, however, we found no local progression during RT or within follow-up (≤12 months). 

Based on our data, irradiation with higher dosages per single fraction over a shorter period of time is sufficient to effectively reduce primary tumor volume. Importantly, radiotherapy-induced acute and late toxicities need to be considered using alternative fractionation regimens [30]. We found a manageable treatment-related toxicity of hypofractionated RT in our single center cohort as well as studies included into systematic review [12,13,14,16,17]. These results, especially of Stavas et al. [12], are similar to our single center data and seem to have more tolerable toxicity profiles than in the previously mentioned studies. On the contrary, toxicity rates obtained in the study of Takahashi et al. [13] excel across the studies included in the systematic review. The authors used an ultra-hypofractionated RT in ATC patients with a median dose per fraction of 5 Gy [13]. Therefore, patients in that study developed acute grade 3 dysphagia, mucositis, and dermatitis in 26%, 5%, and 5% of cases, respectively, in the hypofractionated RT group (*n* = 19), but also one case of grade 4 toxicity due to the injury of trachea and one case of grade 5 injury of carotid artery (18%) were reported. We found that hypofractionated RT (≥4Gy/fr) is not beneficial for OS compared to moderate hypofractionated RT. Importantly, RT with a single dose of 2.50 to 3.50 Gy per fraction shows a trend of more favorable survival compared with ≥4 Gy (12-month survival rate of 23 versus 6%, *p* = 0.077). Currently, the extent to which ultra-hypofractionation is associated with greater toxicity rates is controversial, as it has been reported for several other cancer subtypes [31].

Historically, hyperfractionated RT was considered as an alternative to normo- or hypofractionated RT regimen [32]. Dandekar et al. [10] treated 39 patients (80% with ATC) with hyperfractionated RT and were confronted with higher toxicity rates, when compared to our results: 38%, 12%, 30%, and 30% of grade 3 erythema, desquamation, dysphagia and esophagitis were reported. Respectively, grade 4 toxicity was reported in for 18%, 9%, 44%, and 47% of cases (*n* = 34/39). In addition, local control (complete/partial response and stable disease) was reported in a total of 85% of patients in that research group, which is comparable to the reported results of studies from our systematic review.

With respect to the pathogenesis of radiation-induced toxicities, irradiation dosage may not be the only influence on the rate of adverse events [30]. The actual irradiation technique impacts radiation-induced acute and late toxicities. E.g., IMRT is reported to be safer by delivering higher doses of irradiation (66 Gy vs. 60 Gy 3D-CRT, *p* = 0.005) with a better homogeneity than 3D-CRT, sparing high-risk regions (e.g. salivary gland, myelon) and to have a beneficial impact on OS and progression-free survival (PFS) (OS: HR = 0.30, *p* = 0.005; PFS: HR = 0.33, *p* = 0.005) [33]. Potential escalations are therefore possible because of a lower rate on severe toxicities—a total of 2 patients was reported to develop CTCAE Grade 3 dermatitis after IMRT by Park et al. [33]. In our study, however, IMRT technique did not achieve significance for beneficial OS in the univariate analysis (*p* = 0.701).

Several limitations must be considered for our study such as the retrospective nature and, therefore, a risk of including hidden selection biases. Despite the small patient numbers and long recruiting time in our single center cohort, our pooled analysis remains one of the largest studies reported to date.

Hypofractionated RT shows manageable toxicity with acceptable local control even in dose-escalated regimens. Further prospective studies need to address hypofractionated RT in the context of multimodal treatment of ATC.

## 4. Patients and Methods

### 4.1. Single Center Evaluation

The study was ethically approved by the Institutional Review Board (IRB) of Ludwig-Maximilians University in Munich, Germany (approval number: 20-023). All consecutive patients with histologically confirmed ATC irradiated between 2009 and 2019 were evaluated. Hypofractionated RT to the primary tumor was defined as an irradiation with a single dose of 2.5 Gy or more. Patients receiving palliative radiation with a cumulative dose of ≤30 Gy were excluded. As a result, 11 (32%) patients were irradiated with a hypofractionated regimen and included in the single center cohort (Table 1).

### 4.2. Systematic Review of Literature

A complete literature search was conducted using MEDLINE/Pubmed (National Center for Biotechnology Information, Bethesda, MD, USA) and Cochrane databases on 15 February 2020 in order to identify relevant publications. The search strategy for MEDLINE/PubMed with Boolean operators and applied terms is illustrated in Table 9. All Cochrane reviews concerning ATC were evaluated on 25 February 2020. Data analysis was conducted within a timeframe from 25 February to 2 March 2020. 

Retrospective studies and prospective clinical trials dated from 1 January 2000 to 1 December 2019, written in English and containing search terms shown in Table 9 were included preliminarily. Abstracts were analyzed for eligibility based on irradiation dosage ≥2.5 Gy or hypofractionated RT in palliative or curative situation of ATC. Data regarding performance status and toxicity were extracted and analyzed, if available. Identified systematic reviews meeting search criteria were examined for relevant publications.

Publications reporting RT with a single dose less than 2.5 Gy per fraction were excluded. Pre-clinical in vitro and in vivo studies, drug trials, guidelines, consortia, duplicates, case-reports, and publications without exact specification of fractionation regimen or reviews were excluded. Studies providing no extractable data for groups treated with hypofractionated RT or reporting groups with a lower range of dose per fraction of less than 2.5 Gy were excluded. Flow-chart of literature reviewing process is shown in Figure 2.

### 4.3. Pooled Analysis and Data Management

Eligible publications providing raw data on performance status, disease progression and stage, therapeutic modality, irradiation dosage and OS were extracted and pooled with our single center cohort in order to examine patient characteristics, treatment and outcomes of patients with ATC. Hypofractionated RT was redefined for raw data as ≥2.5 Gy per fraction, thus, aligning treatment specification with the patient cohort and the setting described above. Patients receiving palliative radiation doses with ≤30 Gy (EQD2) were excluded.

Statistical analyses were performed using SPSS statistics 25 (IBM, Chicago, IL, USA). Subgroups were compared using the log-rank test. All significant variables in univariate analysis were included in a multivariate Cox regression analysis. The proportional hazard assumption of the Cox regression analysis was tested. OS was defined as the time between the diagnosis of ATC and death. Patients still alive or lost to follow-up were censored at last visit. For all statistical analyses, a significance level of α = 0.05 was defined.

## 5. Conclusions

Hypofractionated RT appears to achieve sufficient local control with acceptable toxicity. Multimodal treatment and dose escalation (≥50 Gy) are important prognostic factors in patients receiving hypofractioned RT in our single-center cohort and pooled analysis. Hypofractionated radiotherapy appears to be non-inferior compared to normofractionated RT concerning OS. Hypofractioned RT could be an integral part of multimodal treatment and should be investigated in further studies.

## Figures and Tables

**Table 1:** Patient and treatment characteristics of single center cohort.

Parameter	*n*
**Age, years**	
<73	5 (46%)
≥73	6 (55%)
**Gender**	
Male	4 (36%)
Female	7 (64%)
**ECOG-PS**	
0	2 (18%)
1	7 (64%)
2	2 (18%)
**T stage**	
3	1 (9%)
4	10 (91%)
**N stage**	
0	1 (9%)
0	10 (91%)
**M stage**	
0	3 (27%)
1	8 (73%)
**UICC stage**	
IVB	3 (27%)
IVC	8 (73%)
**Surgery**	
No	8 (73%)
Yes	3 (27%)
**Concurrent** **chemotherapy**	
No	4 (36%)
Yes	7 (64%)
**Treatment**	
RT/CRT	8 (73%)
S+CRT	3 (27%)
**EQD2 level**	
<45 Gy	5 (46%)
≥45 Gy	6 (55%)
**RT technique**	
3D-CRT	8 (73%)
IMRT	3 (27%)

ECOG-PS = Eastern Cooperative Oncology Group Performance Score, UICC = Union internationale contre la cancer, IVB/IVC staging according to 8th edition of UICC, RT = radiation therapy, CRT = concomitant chemoradiotherapy, S+CRT = chemoradiotherapy following surgical resection.

**Table 2:** Uni- and multivariate analysis of overall survival (OS) in the single center cohort.

Parameter	At 3	At 6	At 9	*p*-Value	*p*-Value
	Months	Months	Months	(Univariate Analysis)	(Multivariate Analysis)
**Age, years**					
<73	60%	40%	40%	0.106	
≥73	50%	17%	0%		
**Gender**					
Male	71%	57%	43%	**0.04**	0.349
Female	25%	0%	0%		
**ECOG-PS**					
0	100%	50%	50%		
1	57%	43%	29%	0.326	
2	0%	0%	0%		
**M stage**					
0	100%	67%	67%	0.179	
1	38%	25%	13%		
**Treatment**					
RT/CRT	38%	13%	0%	**0.006**	0.941
S+CRT	100%	100%	100%		
**Concurrent** **chemotherapy**					
No	57	29	14	0.327	
Yes	50	50	50		
**EQD2 level**					
< 45 Gy	50%	33%	17%	0.241	
≥ 45 Gy	60%	40%	40%		
**RT technique**					
3D-CRT	50%	38%	38%	0.701	
IMRT	67%	33%	0%		

ECOG-PS = Eastern Cooperative Oncology Group Performance Score, UICC = Union internationale contre la cancer, IVA/IVB/IVC staging according to 8^th^ edition of UICC, RT = radiation therapy, CRT = concurrent chemoradiotherapy, S+CRT = chemoradiotherapy following surgical resection, EQD2 = equivalent dose in 2Gy per fraction.

**Table 3:** Characteristics of patient cohorts and treatment modalities in the systematic review.

Author	Number of Patients (*n*)	Median Age (Years)	Presence of Metastases	Stage Distribution IVA/IVB/IVC (%)	hypoRT in (*n*) Patients	Surgery	ChT	Detailed RT Information
Goutsouliak et al. (2004) [16]	62 referred cases: 57: received radiotherapy, 33 in palliative intention	84 (*n* = 62)	16% (*n* = 62)	NR/NR/16 (*n* = 62)	49	21.0% (*n* = 62)	14.5% (*n* = 62)	(1) *n* = 33: 30 Gy/3 Gy (2) *n* = 8: 50 Gy/2.5 Gy (3) *n* = 5: 40 Gy/2.67 Gy (4) *n* = 2: 45 Gy/2.5 Gy (5) *n* = 1: 45 Gy/3 Gy
Wang et al. (2006) [17]	47: 24 in palliative intention	70.5 (46.1–89.7) (*n* = 24)	25% (IVC)	8/67/25 (*n* = 24)	24	37.5%	16.7%	Median 20 Gy/4Gy (5–40Gy/4Gy)
Stavas et al. (2014) [12]	17	70 (59–84)	47% (*n* = 17)	41/12/47 (*n* = 17) AJCC 7th edition	17	82.4%	88.2%	Median 54Gy/3Gy (40–62.5Gy/2.5–4Gy)
Nachalon et al. (2015) [14]	26 patients: 12 treated in palliative intention	NR	52% (*n* = 23)	NR/NR/52% (*n* = 12)	23	21.7%	47.8%	(1) resectable & ECOG<2: S+RCT 60–70Gy (*n* = 5) (2) non-resect. & ECOG<2: RCT 70Gy (*n* = 6) (3) M+ & ECOG<2: 50Gy (*n* = 9) (4) M+ & ECOG>2: 30Gy (*n* = 3) (5) no treatment (*n* = 3)
So et al. (2017) [15]	30: 18 treated in palliative intention	78 (63–92, *n* = 14)	50% (*n* = 14)	21/29/50 (*n* = 14)	14	28.6%	14.3%	Median 45 Gy/3 Gy (18–45Gy/2.5–6Gy)
Takahashi et al. (2018) [13]	33	71 (49–87) (*n* = 25)	40% (IVC)	19/32/40 (other 1.2%)	25	28.0%	48.0%	Median 50Gy/5Gy (5–60Gy/3–6Gy)

NR = not reported, hypoRT = hypofractionated radiotherapy, IVC = UICC (Union international contre la cancer stage according to 7th edition), ECOG = Eastern Cooperative Oncology Group performance score, AJCC = American Joint Committee on Cancer.

**Table 4:** Outcome of anaplastic thyroid carcinoma (ATC) patients undergoing hypofractionated radiotherapy (RT).

Author	Median FU (95% CI) in Months	Survival after 3 Months	Survival after 6 Months	Survival after 12 Months	Median Survival (95% CI) in Months
Goutsouliak et al. (2004) [15]	3 (0.6–20)	NR	NR	NR	3 (0.6–20) NR for other hypofractionated treatment modalities
Wang et al. (2006) [16]	4.7 (0.2–114)	54.2%	16.7%	0%	3.2 (0.2–NR, <9)
Stavas et al. (2014) [12]	9.3 (4.6–14)	94.1%	70.6%	41.2%	9.3 (4.6–14)
Nachalon et al. (2015) [14]	6 (2.1–9.8)	NR	NR	NR	6 (2.1–9.8)
So et al. (2017) [17]	3.4 (1.9–4.9)	57.1%	21.4%	7.1%	3.4 (1.9–4.9)
Takahashi et al. (2018) [13]	3 (2.5–3.5)	42.9%	23.8%	4.8%	3 (2.5–3.5)

NR—not reported.

**Table 5:** Acute Common Terminology Criteria for Adverse Events (CTCAE) and Radiation Therapy Oncology Group (RTOG) ≥ grade 3 adverse events due to the hypofractionated treatment.

Author	CTCAE/RTOG ≥ Grade 3 Events						Local Recurrence
	Dysphagia	Dyspnea	Dysphonia	Mucositis	Dermatitis	Other Symptoms or Supportive Interventions	
Goutsouliak et al. (2004) [15]	NR	NR	NR	NR	NR	NR	NR
Wang et al. (2006) [16]	NR	NR	NR	0	0	4.2% esophagitis	20.1%
Stavas et al. (2014) [12]	24%	NR	NR	NR	24%	18% esophagitis; 23.5% PEG post-RT	18%
Nachalon et al. (2015) [14]	0	0	0	0	0	23%: PEG 35%: tracheostomy	NR
So et al. (2017) [17]	0	0	0	0	0	0	29%
Takahashi et al. (2018) [13]	26%	NR	NR	5%	5%	10% tracheal necrosis & injury to carotid artery	28% 5% died from LR

NR = not reported, PEG = percutaneous endoscopic gastrostomy, LR = local recurrence.

**Table 6:** Patient and treatment characteristics of pooled patient cohort.

Parameter	*n*
**Age, years**	
<73	30 (50%)
≥73	30 (50%)
**Gender**	
Male	31 (52%)
Female	29 (48%)
**ECOG-PS**	
0–1	35 (58%)
2–4	25 (42%)
**UICC stage**	
IVA	6 (10%)
IVB	22 (37%)
IVC	30 (50%)
unknown	2 (3%)
**EQD2 level**	
<50 Gy	24 (40%)
≥50 Gy	36 (60%)
**Single dose**	
2.5–3.5 Gy	40 (67%)
4–5 Gy	20 (33%)
**Concurrent chemotherapy**	
No	23 (38%)
Yes	37 (62%)
**Treatment**	
RT	24 (40%)
CRT	11 (18%)
S + CRT	25 (42%)

ECOG-PS = Eastern Cooperative Oncology Group Performance Score, UICC = Union internationale contre la cancer; IVA/IVB/IVC staging according to 8^th^ edition of UICC, RT = radiation therapy, EQD2 = equivalent dose in 2Gy per fraction, CRT = concurrent chemoradiotherapy, S+CRT = chemoradiotherapy following surgical resection.

**Table 7:** Uni- and multivariate analysis of overall survival (OS) of the pooled patient cohort.

Parameter	At Three Months	At Six Months	At 12 Months	*p*-Value (Univariate Analysis)	*p*-Value (Multivariate Analysis)
**Age, years**					
<73	77%	58%	19%	0.068	
≥73	62%	35%	15%		
**Gender**					
Male	76%	50%	8%	0.743	
Female	62%	41%	24%		
**ECOG-PS**					
0–1	77%	49%	15%	0.95	
2–4	59%	41%	30%		
**UICC stage**					
IVA/B	74%	51%	25%	0.119	
IVC	67%	42%	11%		
**EQD2 level**					
<50 Gy	50%	33%	8%	**0.014**	**0.065**
50 Gy	82%	54%	24%		
**Single dose**					
2.5–3.5 Gy	73%	51%	23%	0.077	
4–5 Gy	62%	34%	6%		
**Concurrent chemotherapy**					
No	73%	44%	11%	0.286	
Yes	67%	47%	20%		
**Treatment**					
RT/CRT	52%	24%	12%	**<0.001**	**0.003**
S+CRT	92%	68%	32%		

ECOG-PS = Eastern Cooperative Oncology Group Performance Score, UICC = Union internationale contre la cancer; IVA/IVB/IVC staging according to 8th edition of UICC, RT = radiation therapy, EQD2 = equivalent dose in 2Gy per fraction, CRT = concurrent chemoradiotherapy, S+CRT = chemoradiotherapy following surgical resection.

**Table 8:** Patient and treatment characteristics of propensity score matching (PSM) cohort.

Parameter	Entire PSM-Cohort N (%)	Normofractionated Subgroup N (%)	Hypofractionated Subgroup N (%)	*p*-Value
Total	54 (100)	18 (33)	36 (67)	
Age, years (range)	70 (54–86)	68 (55–83)	71 (54–86)	0.235
Gender				
Male	27 (50)	9 (50)	18 (50)	0.999
Female	27 (50)	9 (50)	18 (50)	
ECOG				
0–1	48 (89)	16 (89)	42 (89)	0.999
2–4	6 (11)	2 (11)	4 (11)	
UICC stage				
IVA	4 (10)	2 (11)	2 (6)	
IVB	23 (37)	13 (72)	10 (28)	**0.002**
IVC	26 (50)	3 (17)	23 (64)	
EQD2 level				
<50 Gy	14 (26)	2 (11)	12 (33)	0.082
≥50 Gy	40 (74)	16 (89)	24 (67)	
Concurrent chemotherapy				
No	18 (33)	6 (33)	12(33)	0.999
Yes	36 (67)	12 (67)	24 (67)	
Treatment				
RT/CRT	26 (48)	6 (33)	20 (56)	0.128
S+CRT/RT	28 (52)	12 (67)	16 (45)	

**Table 9:** Search terms used for PubMed/MEDLINE database search.

Term		Studies Identified
1	(Radiotherapy or radiotherap* or radio-therap* or irradiation or irradiat* or re-irradiat* or reirradiat*)	530,114
2	(“anaplastic thyroid cancer” or “anaplastic thyroid carcinoma” or ATC)	4658
3	1 and 2	333
4	“2000/01/01” [PDat]: “2019/12/01” [PDat]	267
